# Fabrication of Cellulose Acetate-Based Proton Exchange Membrane with Sulfonated SiO_2_ and Plasticizers for Microbial Fuel Cell Applications

**DOI:** 10.3390/membranes13060581

**Published:** 2023-06-02

**Authors:** Gowthami Palanisamy, Yeong Min Im, Ajmal P. Muhammed, Karvembu Palanisamy, Sadhasivam Thangarasu, Tae Hwan Oh

**Affiliations:** 1 School of Chemical Engineering, Yeungnam University, Gyeongsan 38541, Republic of Korea; yym2150@naver.com (Y.M.I.); ajmalpm127@gmail.com (A.P.M.); sadhasivam.nano@gmail.com (S.T.); 2 Department of Microbiology, Punjab Agricultural University, Ludhiana 141004, Punjab, India; karvembu007@gmail.com

**Keywords:** proton exchange membrane, microbial fuel cell, cellulose, cellulose acetate, hybrid polymer composite, inorganic SiO_2_ filler, plasticizer, sulfonated SiO_2_

## Abstract

Developing a hybrid composite polymer membrane with desired functional and intrinsic properties has gained significant consideration in the fabrication of proton exchange membranes for microbial fuel cell applications. Among the different polymers, a naturally derived cellulose biopolymer has excellent benefits over synthetic polymers derived from petrochemical byproducts. However, the inferior physicochemical, thermal, and mechanical properties of biopolymers limit their benefits. In this study, we developed a new hybrid polymer composite of a semi-synthetic cellulose acetate (CA) polymer derivate incorporated with inorganic silica (SiO_2_) nanoparticles, with or without a sulfonation (–SO_3_H) functional group (sSiO_2_). The excellent composite membrane formation was further improved by adding a plasticizer (glycerol (G)) and optimized by varying the SiO_2_ concentration in the polymer membrane matrix. The composite membrane’s effectively improved physicochemical properties (water uptake, swelling ratio, proton conductivity, and ion exchange capacity) were identified because of the intramolecular bonding between the cellulose acetate, SiO_2_, and plasticizer. The proton (H^+^) transfer properties were exhibited in the composite membrane by incorporating sSiO_2_. The composite CAG–2% sSiO_2_ membrane exhibited a higher proton conductivity (6.4 mS/cm) than the pristine CA membrane. The homogeneous incorporation of SiO_2_ inorganic additives in the polymer matrix provided excellent mechanical properties. Due to the enhancement of the physicochemical, thermal, and mechanical properties, CAG–sSiO_2_ can effectively be considered an eco-friendly, low-cost, and efficient proton exchange membrane for enhancing MFC performance.

## 1. Introduction

Microbial fuel cell technologies (MFCs), which are new innovative green-energy technologies, have attracted attention over their simultaneous wastewater treatment and bioelectricity generation through their biological and electrochemical properties [1,2,3]. In MFCs, the electrons and protons are generated from the anaerobic digestion of organic effluents by electrogens in the anode chamber. The generated electrons are transferred through the external circuit, while the generated protons are transported through the proton exchange membrane (PEM) to the cathode chamber to complete redox processes and generate bioelectricity [4,5]. PEM is the key component in MFCs, which acts as a barrier between the cathode and anode chambers. It affords proton transport channels for transferring protons from the anode to the cathode chamber. It also prevents the effluent, oxygen, and proton crossover from the cathode to the anode chamber. An ideal PEM in MFCs should be cost-effective and have enhanced mechanical and chemical stability, a high proton conductivity, biofouling resistivity, etc. A commercially available perfluorosulfonic acid polymer membrane, namely Nafion (Dupont), is the most commonly used PEM due to its increased proton conductivity in MFCs. Nevertheless, the non-biodegradability, increased fuel crossover and substrate loss, and poor thermal stability have led to a quest for a low-cost, high-efficiency replacement membrane material [5,6]. Nonetheless, even though MFCs are evolving as viable, environmentally-friendly alternatives to conventional energy-generating technologies, the membrane for MFCs relies on synthetic polymeric materials such as sulfonated poly(ether-ether-ketone)s (SPEEKs), sulfonated polybenzothiazoles (SPBOs), sulfonated polysulfone (SPSU), polyvinylidene difluoride (PVDF), polyvinyl alcohol (PVA), sulfonated polybenzimidazole (SPBI), sulfonated poly(ether sulfone) (SPES), sulfonated poly (arylene-ether-ketone)s (SPAEKs), and sulfonated poly(arylene-ether-nitrile)s (SPAENs), which have been extensively used in past years [7,8,9,10,11]. On the other hand, each of these materials has its own benefits and drawbacks, and research is underway to identify the most cost-effective and ideal membrane for MFC applications.

In recent years, biopolymer-based membranes have gained great attention as membrane candidates in MFCs due to their intrinsic features, such as their cost-effectiveness, biocompatibility, eco-friendliness, and natural abundance. As a result, different kinds of biopolymers, such as cellulose and its derivatives, chitosan, alginate, and lignocellulosic materials, have been used as host polymeric materials for fabricating PEMs. Among these, cellulose stands out as a natural biopolymer that is ideal for proton conduction due to its low density, biodegradability, renewability, biocompatibility, high mechanical strength, and improved intra- and intermolecular hydrogen bonding capabilities [12,13]. Recently, cellulose derivatives, such as cellulose acetate (CA), have garnered considerable interest for their economic viability, availability, eco-friendliness, and modifiability [14,15]. Cellulose acetate is an ester of cellulose produced by the partial or complete acetylation of the free hydroxyl groups in the anhydrous glucose unit. Additionally, it has a great affinity towards positively charged protons, because it possesses negatively charged polysaccharides on its backbone structure [16]. Nevertheless, unmodified CA has a low ion exchange capacity, indicating its poor proton conductivity, which renders it unsuitable for use as a membrane in fuel cells. In order to rectify these drawbacks, many researchers have created CA-based membranes for fuel cell applications by either introducing inorganic fillers/additives or functional groups to the CA polymer or by fabricating it with another proton-conductive polymer, thereby expanding its potential as a proton exchange membrane (PEM) in fuel cells [17,18,19,20,21,22]. Henceforth, many studies have incorporated inorganic fillers such as silicon dioxide (SiO_2_), graphene oxide (GO), zirconium dioxide (ZrO_2_), titanium dioxide (TiO_2_), and carbon nanotubes (CNTs) into the membrane matrix for enhancing the membrane performance. Thus, an inorganic filler-incorporated polymer composite membrane exhibits enhanced PEM properties due to its chemical reactivity, mechanical and thermal stability along with a reduction in the decomposition of the polymer of the inorganic backbone, and flexibility of the organic polymer backbone [18,23,24,25]. Among different inorganic fillers, silica was selected for its cost-effectiveness along with its enhanced physical, chemical, and thermal properties and a large surface area [5]. Here, silica incorporation into the polymer matrix enhanced the ionic conductivity of the composite membrane, which was attributed to the high water retention properties of silica [26]. Additionally, the functionalization of SiO_2_ with sulfonic acid groups in the composite membrane resulted in a higher proton conductivity than the composite membrane with unsulfonated SiO_2_. The reason involves the –SO_3_H groups in the sulfonated SiO_2_ developing a proton transfer path through ionic clusters (water-conducting channels), and with its negative charges, it itself acts as a carrier vehicle [27,28,29]. Moreover, sulfonated SiO_2_ in the composite membrane decreases the glass transition temperature (T_g_) and enhances the polymer’s amorphous properties [30]. It has been observed that adding a plasticizer to a polymer matrix reduces the energy required for molecular motion and develops hydrogen bonds between polymer chains. Additionally, plasticizers also create a large amount of “free-volume” space in the composite membrane that absorbs water, which facilitates the increment in proton conductivity [31]. Thus, plasticizers have the ability to increase the membrane flexibility and remove membrane shrinkage and frailty [24]. Here, glycerol was incorporated into the polymer matrix for enhancing the membrane’s proton conductivity and flexibility and allowing for a high water uptake due to its free-space volume as well as its hygroscopic properties.

The primary objective of this study was to fabricate a composite membrane (CA–gly–sSiO_2_) with cellulose acetate (CA) as the fundamental material and glycerol and sulfonated inorganic SiO_2_ filler to enhance the membrane’s performance in a microbial fuel cell (MFC) configuration. Here, the effects of glycerol, unsulfonated SiO_2_, and sulfonated SiO_2_ on the composite membranes, such as effects on the surface morphology, mechanical stability, and thermal behavior, were examined through a sophisticated analysis. Additionally, the membrane’s physicochemical properties, namely its water uptake, swelling ratio, hydrophilic and hydrophobic properties, proton conductivity, and ion exchange capacity, were investigated. It was observed that the incorporation of sulfonated SiO_2_ into the composite membrane resulted in enhanced structural, thermal, mechanical, and physicochemical properties, which led to an improved MFC performance.

## 2. Materials and Methods

### 2.1. Materials

Cellulose acetate and SiO_2_ were purchased from Sigma-Aldrich. Glycerol was procured from Acros Organics. Sulfuric acid (H_2_SO_4_), sodium hydroxide (NaOH), and sodium chloride (NaCl) were obtained from the Duksan reagent. Acetone and N, N-dimethylformamide were purchased from Daejung Chemicals (Siheung-si, Republic of Korea). The chemicals and reagents utilized in this experiment were of analytical grade, and further purifications were unnecessary.

### 2.2. Preparation of Sulfonated SiO_2_

The sulfonation of SiO_2_ was performed according to previously described procedures [28,30]. To begin with, any impurities in the SiO_2_ were removed by drying it in a furnace at 500 °C [32]. Afterwards, 30 mL of ethanol was added into a predetermined amount of SiO_2_ and ultra-sonicated for 30 min. This step aimed to introduce the hydroxyl (–OH) functional groups into SiO_2_ [33]. Following this, the desired amount (1 g) of SiO_2_ was introduced into 20 mL of a methanol solution containing 15 mL of 0.5 M sulfuric acid, and the solution was placed under ultrasonication for 60 min. Sulfonated SiO_2_ was produced as the end product after the solution mixture had been evaporated at 100 °C.

### 2.3. Membrane Fabrication

**Figure 1:**
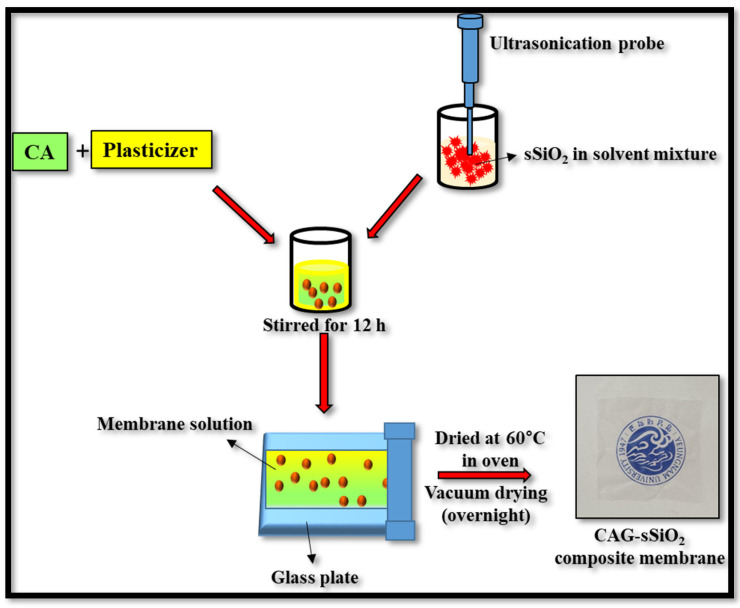
Schematic illustration of CAG–sSiO_2_ composite membrane preparation.

Pure cellulose acetate (CA) and the CA composite membranes incorporated with glycerol, SiO_2_, and sulfonated SiO_2_ were prepared using a solution-casting technique. For preparing a pristine CA membrane, 15% *w*/*v* of CA was dissolved in an acetone and DMF mixture (2:1) and the solution was kept under magnetic stirring for 24 h [34]. Then, the obtained homogenous solution was cast on a clean, dry glass plate using a 300 µm casting knife. The glass plate was then dried at 60 °C for 12 h to obtain the pristine CA membrane. To prepare the CAG membrane, 30% glycerol was added to the polymer solution, and the membranes were cast in the same way as the pristine CA membrane. To prepare the inorganic composite membranes, the desired proportions of pure SiO_2_ (1 wt%) and sSiO_2_ (0.5%, 1%, or 2 wt%) were uniformly dispersed into the solvent mixture (acetone: DMF mixture) through ultrasonication. The desired amount of CA and glycerol were introduced into the suspension and continuously stirred for 24 h. At last, the homogenized solution was cast on a clean glass plate using a casting knife and then dried in a vacuum oven (Figure 1). The dried membrane was then denoted as CAG–SiO_2_, CAG–0.5% sSiO_2_, CAG–1% sSiO_2_, or CAG–1% sSiO_2_.

### 2.4. Membrane Characterization

Fourier-transform infrared (FT-IR) spectroscopy (Perkin Elmer, Spectrum 100, Waltham, MA, USA) was used to analyze the membrane’s functional groups. During the analysis, the measurements of the spectra were performed at the transmittance mode in the range of 4000–600 cm^−1^. The membrane’s structural characteristics were analyzed through an X-ray diffraction (XRD) analysis, namely PANalyticalX’pert, employed with Cu Kα radiation of wavelength 1.540 Å. A field-emission scanning electron microscope (FE-SEM) (Hitachi, S-4800, Tokyo, Japan) was used for analyzing the structural and elemental composition of the membrane.

A thermogravimetric analysis of the film samples was performed using an SDT Q600 (simultaneous DSC-TGA) instrument. Approximately 5 mg of film samples were heated in a standard ceramic crucible from 30 °C to 600 °C at a 10 °C heating rate. The analysis was conducted in a nitrogen atmosphere at a constant purge rate of 200 mL/min.

#### Mechanical Properties

The tensile strength (TS) and elongation at break (EAB) of the film samples were measured by following the ASTM standard method D882 using a universal testing machine (3345, INSTRON, Norwood, MA, USA). The film samples were cut precisely into strips of 60 × 10 mm dimensions using a sharp cutting knife. The analysis was performed at an initial grip separation of 30 mm and a crosshead speed of 5 mm/min.

The water contact angle (WCA) of the prepared film samples was analyzed using a contact angle analyzer (OCA 20 analyzer, Dataphysics, Republic of Korea). The samples were cut into 1.5 × 1 cm rectangular specimens and attached to a movable steel stage on the analyzer. Approximately 10 μL of DI water was allowed to fall on the film surface using a micro syringe, and the WCA measurement was performed immediately.

The water uptake (WU) and swelling ratio (SR) were measured based on changes in the weight and area. Initially, the membrane samples were cut into 4 cm × 4 cm pieces and dried in an oven at 50 °C for 24 h. The membrane’s dry weight and area (*W_dry_* and *A_dry_*) were measured immediately after drying the samples. Then, the membrane samples were soaked in DI water for 24 h, and then water on the membrane surface was removed using filter paper. Afterwards, the hydrated membrane’s weight and area (*W_wet_* and *A_wet_*) were measured. The equation used for calculating the membrane water uptake (WU%) is described below:

$$\mathrm{Water}\;\mathrm{uptake}\;\left(\%\right)=\frac{W_{wet}-W_{dry}}{W_{dry}}\times 100$$

whereas the membrane swelling ratio was calculated by a change in the area as follows:

$$\mathrm{Swelling}\;\mathrm{ratio}\;\left(\%\right)=\frac{A_{wet}-A_{dry}}{A_{dry}}\times 100$$

A titration method was performed to determine the membrane’s ion exchange capacity (IEC). The dried membrane was measured and then soaked in 100 mL of a 1 M NaCl solution for 24 h to enable the replacement of all H^+^ sites with Na^+^ ions. Then, the membrane was removed and the remaining solution, containing protons released from the membranes, was titrated against a 0.01 M NaOH solution using phenolphthalein as an indicator. The following equation was used for calculating the IEC:

$$\mathrm{Ion}\;\mathrm{exchange}\;\mathrm{capacity}\;\left(\mathrm{meq}/g\right)=\frac{V_{NaOH}\times C_{NaOH}}{W_{dry}}$$

Here, the NaOH solution’s concentration was denoted by $C_{NaOH}$, the volume of the NaOH solution used during the titration was denoted as $V_{NaOH}$, and the dry weight of the membrane was denoted by $W_{dry}$.

The membrane’s proton conductivity (σ) was calculated using electrochemical impedance spectroscopy from 100 mHz to 1 MHz via a Corrtest instrument. Here, the membrane was cut into 1 cm × 6 cm pieces and placed in a Teflon cell containing stainless steel electrodes in DI water for a conductivity measurement. The proton conductivity with the measured resistance was calculated using the following equation:

$$\mathrm{Proton}\;\mathrm{conductivity}\;\left(S/\mathrm{cm}\right)=\frac{D}{R\times A}$$

where *D* is the distance between two electrodes (cm), *R* is the resistance calculated from the Nyquist impedance plot (Ω), and *A* is the membrane’s effective area.

## 3. Results and Discussion

**Figure 2:**
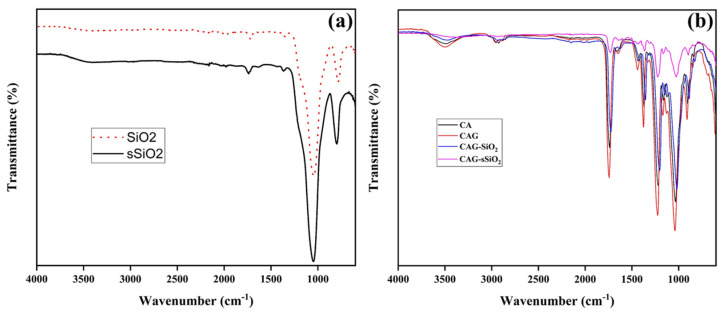
FTIR spectra of (**a**) SiO_2_ and sSiO_2_ and (**b**) pristine and composite CA membranes.

The FTIR spectra of pure SiO_2_ and sulfonated SiO_2_ (sSiO_2_) particles were obtained to investigate their chemical composition, and they are shown in Figure 2a. From the spectra, the highest adsorption at 1049 cm^−1^ and 801 cm^−1^ relied on Si-O-Si asymmetrical stretching and Si-OH vibrational stretching, respectively [35,36,37]. The peak observed at 1740 cm^−1^ corresponded to the O-H bending vibration of adsorbed water molecules. Meanwhile, the broad peak at 3375 cm^−1^ was attributed to the –OH stretching vibrations of the adsorbed water molecules [28,29,38,39,40]. After sulfonation, the characteristic signal at 3400 cm^−1^ was broadened due to the hydrogen bond broadening the hydroxyl peaks [41]. Figure 2b illustrates the FTIR spectra details of the pristine CA membrane and the CAG, CAG–SiO_2_, and CAG–sSiO_2_ composite membranes. The broad peak in the 3490 cm^−1^ region indicated the O-H stretching vibrations of the pristine CA membrane. This can be seen in all the composite membranes with different intensities. A peak for the C-H asymmetric stretching vibrations was observed at 2921 and 2849 cm^−1^. The characteristic sharp peak at 1736.03 cm^−1^ was assigned to the carbonyl ester stretching vibrations of cellulose acetate, while the peaks at 1431 cm^−1^ and 1366.66 cm^−1^ represented the CH_2_ asymmetric and CH_3_ symmetric angular deformations, respectively. The peaks for the C-O asymmetric stretching of the C-O-O-H and C-O-C of the pyranose rings were observed at 1215 cm^−1^ and 1031 cm^−1^, respectively [42,43]. From the figure, it was observed that the intensity of the peak in the 3490 cm^−1^ region (O-H stretching vibrations) increased due to the presence of glycerol in the CAG composite membrane. This indicated a successful hydrogen bond formation between the polymer (cellulose acetate) hydroxyl groups and the glycerol –OH groups. The identification of structural changes in the composite membranes compared to the pristine CA membrane was difficult due to band overlapping. However, in the CAG–SiO_2_ and CAG–sSiO_2_ composite membranes, the Si-O-Si asymmetrical stretching at 1049 cm^−1^ overlapped with the C-O asymmetric stretching vibration of the CA membrane.

**Figure 3:**
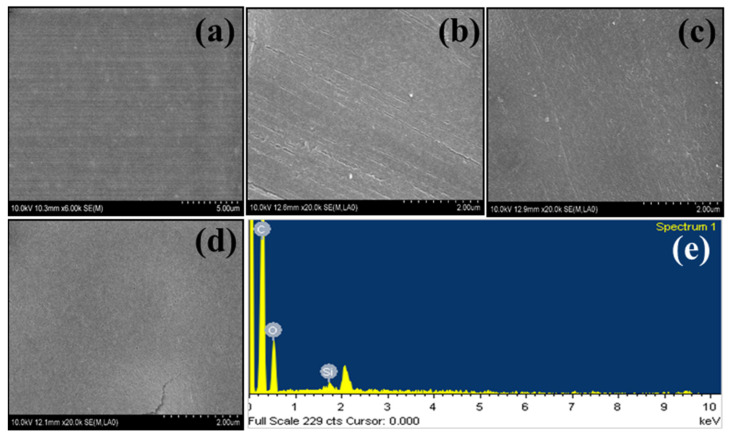
SEM images of (**a**) pristine CA membrane; (**b**) CAG membrane; (**c**) CAG–SiO_2_ membrane; and (**d**) CAG–sSiO_2_ membrane. (**e**) EDX spectra of CAG–SiO_2_ membrane.

Figure 3a–d exhibit the SEM analyses of the pristine CA and composite membranes (CAG, CAG–SiO_2_, and CAG–sSiO_2_). It was observed that the pristine CA membrane displayed a smooth surface. It was clear that the surface roughness was not significantly altered morphologically by the incorporation of fillers into the composite membrane. In the composite membranes, the sulfonated fillers were homogeneously dispersed in the polymer matrix. The uniform dispersion of SiO_2_ in the polymeric matrix as well as the interfacial contact between the filler and polymeric matrix were both improved by the sulfonation of SiO_2_. The EDX analysis of the CAG–SiO_2_ membrane (Figure 3e) confirmed the homogeneous dispersion of SiO_2_ in the polymer membrane matrix.

**Figure 4:**
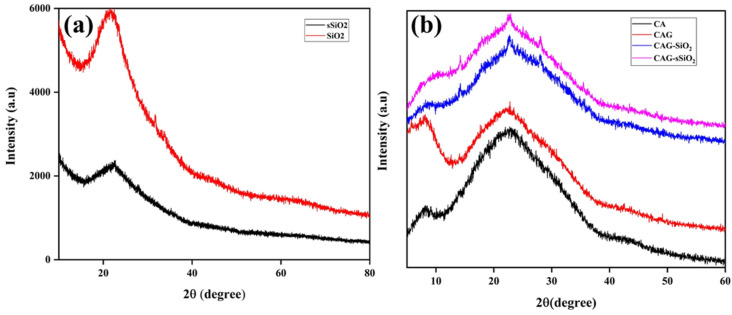
XRD pattern of (**a**) SiO_2_ and sSiO_2_ and (**b**) pristine and composite CA membranes.

The XRD analysis was generally implemented for investigating the morphological characteristics, notably the crystalline behavior, of the composite membranes. Figure 4a depicts the XRD diffraction patterns, illustrating the crystalline behavior of the SiO_2_ and sSiO_2_ particles. The broad peak at 2θ = 22.4° was demonstrated to rely on the peak of SiO_2_. During sulfonation, the sulfonyl group of SiO_2_ reduced the crystallinity and enhanced the amorphous nature of the material [28].

Figure 4b shows the XRD patterns of the pristine CA membrane, the CAG membrane, and the unsulfonated CAG-SiO_2_ and sulfonated CAG-sSiO_2_ composite membranes. The pristine CA membrane showed a peak at the 2θ regions of 8°, 22.8°, and 42°. The peak at 2θ = 8° has been referred to as the characteristic peak of CA, formed by the acetylation of cellulose. During acetylation, the acetyl group disintegrates the cellulose chains, resulting in a subsequent increment in the interfibrillar distance and the destruction of their microfibrillar structure [44,45,46]. The peak at 2θ = 22.8° represents the peak of the cellulose I form. From this, it was observed that the crystalline behavior was not altered during acetylation. The short hump at 2θ = 42° occurred by short-range intramolecular hydrogen bonding. However, it is worth noting that the glycerol addition to the CA membrane decreased its crystalline behavior [47]. The intermolecular H bonds of CA were broken by glycerol, which directly interacted with CA. When glycerol interacted with the polymer chains, the crystalline phase of the CA (micro-crystalline blocks) was interrupted. This was observed through a decrease in the hump intensity in the CAG membrane [48]. From the XRD profile, it was observed that incorporating the sulfonated inorganic filler (sSiO_2_) into the membrane altered the crystalline nature of the composite membrane [29]. The sharp peak for the CAG–SiO_2_ and CAG–sSiO_2_ composite membranes was identified due to its amorphous properties compared to the pristine CA membrane. The composite membrane flexibility and mechanical stability increased with a decrease in the crystallinity (i.e., increase in amorphous properties [49]). This might have been due to the strong coordination between the CA polymer matrix and the sSiO_2_ particles.

**Figure 5:**
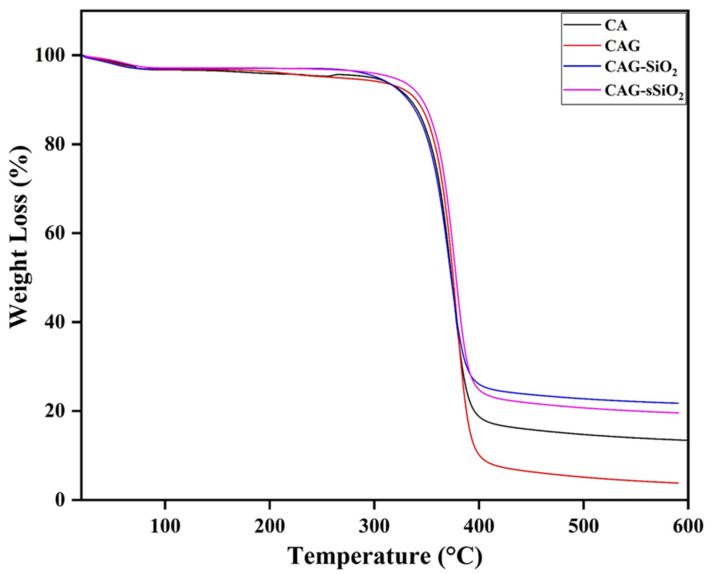
Thermogravimetric analysis of pristine and composite CA membranes.

Figure 5 shows the thermal stability of pristine CA along with that of the CAG, CAG–SiO_2_, and CAG–sSiO_2_ prepared composite membranes. An initial weight loss of around 3.2% was observed below 100 °C in all the membranes, which was due to the evaporation of water and solvent in the membranes [50]. The main thermal degradation of cellulose acetate polymer chains, which represents the decomposition of CO_2_, CO, H_2_O, and acetic acid, was observed in the range of ~300 °C–400 °C [49,51]. Finally, the carbonization of the degraded products started at the temperature of around ~400 °C [52]. It was observed that the incorporation of SiO_2_ into the composite membrane enhanced the thermal stability [53]. This was observed through a higher onset temperature in the composite membrane for weight loss than the pristine CA membrane. The interaction between the –SO_3_H group of filler and the polymer matrix enhanced the thermal stability of the CAG–sSiO_2_ composite membrane. These results explain that incorporating inorganic silica into the polymer matrix enhances the membrane matrix’s strength and improves the thermal behavior.

**Figure 6:**
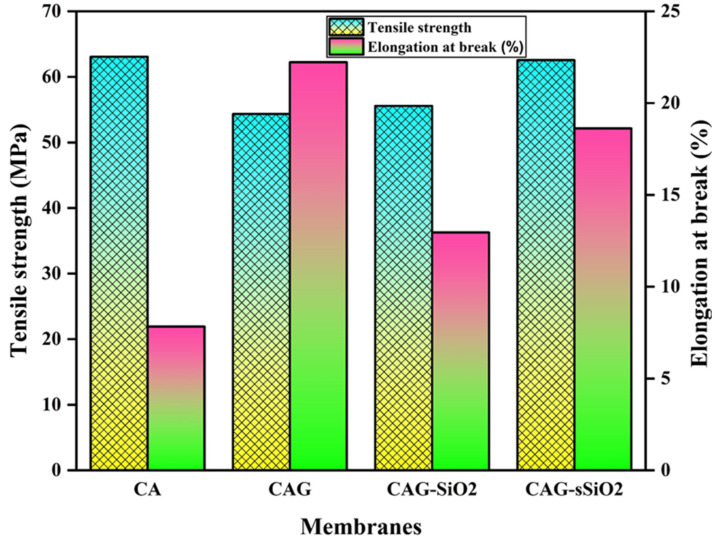
Tensile strength and the elongation at the break of pristine and composite CA membranes.

The tensile strength (TS) and elongation at break (EB) of the pristine CA and composite membranes were determined and are displayed in Figure 6. It was observed that adding glycerol into the cellulose acetate reduced the stiffness of the CAG composite membrane. In the CAG composite membrane, glycerol functions as a plasticizer that interacts with the polymer (CA) network to rearrange the structure, thereby increasing the membrane’s flexibility and decreasing tensile strength [43,54]. The membrane’s flexibility and extensibility are determined by the EB values obtained by maximum stretching before membrane rupturing. It was determined that the CAG composite membrane exhibited higher EB values (22.23%) than the pristine CA membrane due to the presence of the glycerol plasticizer.

Moreover, all the composite membranes exhibited higher EB values than the pristine CA membrane. In the presence of SiO_2_, longer cross chains are formed with the CA polymer network, and thus, the membrane shows membrane folding and unfolding properties based on its higher elasticity (EB value). In the CAG–SiO_2_ composite membrane, the accountable interactions identified between inorganic SiO_2_ fillers and cellulose acetate polymer chains shifted the stress between the polymer matrix and the inorganic SiO_2_ fillers. Thus, higher EB values were exhibited for the CAG–SiO_2_ composite membrane. Compared to all the other membranes, the CAG–sSiO_2_ composite membrane showed a higher tensile strength (62.5624 MPa) with increased elongation-at-break values. Here, the –SO_3_H groups of sSiO_2_ formed hydrogen bonds with –OH groups in the cellulose acetate polymer matrix and, thus, enhanced the CAG–sSiO_2_ composite membrane’s stiffness [28,55]. Moreover, the glycerol in the composite membrane resulted in an enhanced elasticity behavior compared to the composite CAG–sSiO_2_ membrane. Finally, the prepared composite CAG–sSiO_2_ membrane had a more enhanced mechanical strength than the pristine CA membrane, and it has been used as a proton exchange membrane for MFC applications.

The wettability of the prepared composite membranes was quantitatively examined through contact angle measurement using water droplets on the sample surface. It was observed that the water contact angle increased in the CAG–SiO_2_ composite membrane (64.15°) when compared to the pristine CA membrane. This increment may be attributed to the agglomeration of SiO_2_ particles on the membrane’s surface [56]. Meanwhile, the composite CAG–sSiO_2_ membrane (57.5°) retained its membrane hydrophilicity due to the incorporation of sulfonated SiO_2_ into the membrane. This hydrophilic nature was obtained by the presence of a hydrophilic –SO_3_H group in the composite membrane. Hence, a large number of water molecules were retained on the CAG–sSiO_2_ membrane surface by the formation of hydrogen bonds with the water molecules. Thus, a decrease in the water contact angle was observed for the composite CAG–sSiO_2_ membrane.

**Figure 7:**
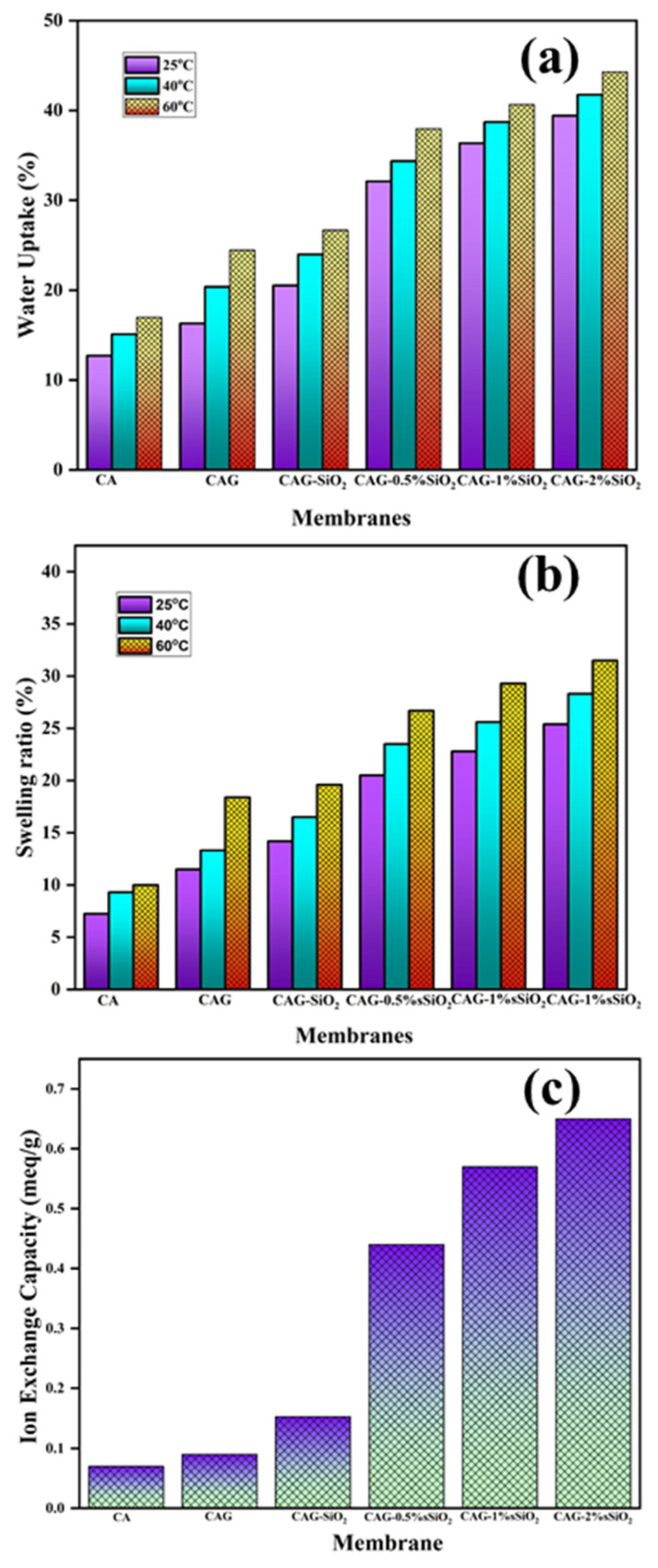
(**a**) Water uptake, (**b**) swelling ratio, and (**c**) IEC of the pristine and composite CA membranes.

Water molecules in addition to hydrogen-bonded ions act as proton carriers in the membrane, thereby ensuring adequate membrane conductivity. However, the membrane experiences mechanical disintegration when its water uptake levels are above optimal. Meanwhile, increasing the temperature improves the mobility of polymer chains, resulting in more space available for water absorption. Figure 7a shows the increment in the water uptake values for the composite membranes due to the incorporation of glycerol and inorganic sulfonated SiO_2_ fillers with an increase in temperature. It was observed that, by introducing glycerol into cellulose acetate, the glycerol occupies more free space between the polymer chains and generates free volume for water uptake in the composite CAG membrane. The hydrophilic properties were also improved, as the surface hydroxyl groups (–OH) of the SiO_2_ filler and the sulfonic acid groups (–SO_3_H) of the sSiO_2_ particles formed hydrogen bonds or electrostatic bonds between the polymer and water, leading to the membrane retaining attractive water uptake properties [53]. Moreover, the increased water uptake properties enhanced the membrane’s proton conductivity by ionizing its sulfonic acid moieties. Among different composite membranes, the CAG–2% sSiO_2_ membrane exhibited the highest water uptake value (44.9% at 60 °C) due to the presence of sulfonic acid groups. Similarly, the membrane swelling ratio and water uptake are relevant to each other. It can be observed from Figure 7b that the presence of hydrophilic sSiO_2_ particles in the CAG–sSiO_2_ composite membrane led to higher swelling ratio values. This was attributed to a higher intake and holding of water molecules by hydrophilic sSiO_2_ molecules. Furthermore, the IEC of a composite membrane relies on the number of acidic groups on the polymer matrix, which are responsible for proton transportation in the membrane matrix. It was shown that incorporating sulfonated SiO_2_ into the membrane matrix increased the IEC of the CAG–sSiO_2_ composite membrane with increased sSiO_2_ proportions (Figure 7c). Here, in the composite membrane, the –SO_3_H group enhanced the ion exchange properties by creating more charge sites with a reduced resistance [57]. As a result, the composite membrane’s proton conductivity increased due to an increased water uptake and increased IEC values.

**Figure 8:**
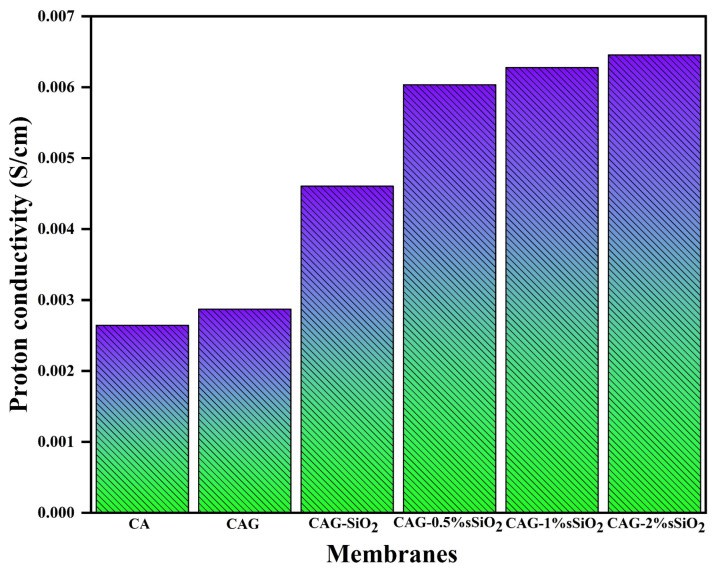
Proton conductivity of the pristine and composite CA membranes.

The proton conductivity is considered the most significant membrane property for MFC performance. Figure 8 presents the proton conductivity values of pristine CA and all the composite membranes. The composite membranes exhibited higher proton conductivity values than the pristine CA membrane (0.002645 S/cm). The improvement in the proton conductivity was attributed to the alteration in the polymer chain mobility [16]. The presence of glycerol in the co”posi’e membrane enhanced the proton conductivity by reducing the crystalline phase and increasing the amorphous region in the membrane [58]. Thus, it enhanced the membrane’s proton conductivity by elevating the intra- and inter-chain movements in the polymer [59]. Moreover, the intermolecular interaction between the glycerol (H atoms) and the polymer chain (oxygen atoms) also increased the polymer chain’s flexibility, to which the ionic conductivity enhancement was attributed [60]. On one hand, the incorporation of SiO_2_ into the membrane resulted in a higher proton conductivity than that of the pristine GA membrane due to increased water uptake properties by its hygroscopic property. On the other hand, the sulfonated SiO_2_ composite membrane exhibited a higher proton conductivity than all the other composite membranes. During the impedance analysis, the sulfonated composite membranes (CAG–0.5% SiO_2_, CAG–1% SiO_2_, and CAG–2%SiO_2_) exhibited lower membrane resistance. Hence, through the Nyquist plot analysis, the proton conductivities were derived and found to be 0.006 S/cm, 0.0063 S/cm, and 0.0065 S/cm for the CAG–0.5% sSiO_2_, CAG–1% sSiO_2_, and CAG–2% sSiO_2_ composite membranes, respectively. Here, the presence of proton-conducting sulfonic acid groups (–SO_3_H groups) on the surface of SiO_2_ improved the charge-carrying sites in the polymer membrane matrix, which generated more proton transport channels for increasing the proton conductivity [27]. Additionally, the sulfonated SiO_2_ particles in the composite membrane held the water molecules through their chemical and physical morphology, which aided in enhancing the proton conductivity. Moreover, the –SO_3_H groups in the SiO_2_ created an excellent ionic bundle inside the composite membrane and enhanced the water absorption properties, thereby enhancing the proton conductivity. The increased water absorption of the composite membrane also generated H_3_O^+^ ions, which resulted in additional proton conduction channels through a hopping mechanism to an adjacent water molecule [30]. Additionally, the –SO_3_H groups in the CAG–sSiO_2_ composite membrane relied on the movement of ionizable groups, which helped with proton conduction. As a result, it was seen that the CAG–2% sSiO_2_ composite membrane exhibited higher proton conductivity than all the other membranes in this present investigation. Similar to previously reported investigations [5,27,28,29,61,62,63,64,65,66] (Table 1), this study also implied that the presence of SiO_2_ in the polymer matrix can effectively influence the membrane performance. Thus, it can be concluded that the CAG–sSiO_2_ composite membrane can be considered an excellent membrane candidate for various energy and environmental systems.

## 4. Conclusions

In this study, a composite proton exchange membrane consisting of a cellulose acetate polymer matrix, incorporated with glycerol as a plasticizing agent and inorganic sulfonated silica, was fabricated through a solution-casting technique for MFC applications. The composite membranes with inorganic sulfonated additives were characterized through FTIR, XRD, SEM, and TGA analyses. The physicochemical properties of pristine CA, CAG, CAG–SiO_2_, and CAG–sSiO_2_ composite membranes were analyzed. It was observed that the CAG–2%sSiO_2_ composite membrane exhibited a higher tensile strength along with an enhanced elasticity. This was attributed to the presence of glycerol and sulfonated silica in the composite membrane. Along with the membrane’s mechanical strength, the water retention property of the composite membrane was also increased. Moreover, the CAG–2% sSiO_2_ composite membrane showed the highest proton conductivity, a higher IEC, and higher water uptake values than the other membranes. This enhancement resulted from the incorporation of inorganic sulfonated SiO_2_ along with a plasticizer. The –SO_3_H groups in the composite membrane generated more ion transport channels that enhanced the ionic mobility, resulting in an improved MFC performance. Finally, the present study discovered that the addition of plasticizers and sulfonated inorganic additives into the composite membrane can effectively enhance the MFC performance.

## Figures and Tables

**Table 1:** Comparative performance of different kinds of SiO_2_-containing membranes.

Membrane	Proton Conductivity	Elongation at Break (%)	Ref.
NMPC/PVA-SiO_2_ (4 wt%)	0.508 mS/cm at 100 °C		[61]
sPEAK/f-SiO_2_-18	8.5 mS/cm	10.7	[62]
sGO@SiO_2_/PVDF-g-PSSA	78 mS/cm		[27]
SM-SiO_2_ + SPPSU	5.9 mS/cm 80 °C and 50% RH		[63]
CL-sPAEK/silica	3.06 mS/cm, at 70 °C under 30% RH	5.2	[64]
80 wt% sPEEK-20 wt% sPVdF-HFP-06 wt% sSiO_2_	79 mS/cm at RT	35.8	[65]
sPEEK/S-SiO_2_/MOF-5	3.69 mS/cm	4.00	[28]
80 wt% sPEEK–20 wt% PVdF-HFP+7.5% SiO_2_	0.08 mS/cm		[5]
sPEEK–7.5% sSiO_2_	10.18 mS/cm		[29]
sPEEK+7.5 wt% sSiO_2_	12.4 mS/cm		[66]
CAG–2% sSiO_2_	6.5 mS/cm	22.23	This study

## Data Availability

Not applicable.
